# Metformin Increases Survival in Hypopharyngeal Cancer Patients with Diabetes Mellitus: Retrospective Cohort Study and Cell-Based Analysis

**DOI:** 10.3390/ph14030191

**Published:** 2021-02-26

**Authors:** Yung-An Tsou, Wei-Chao Chang, Chia-Der Lin, Ro-Lin Chang, Ming-Hsui Tsai, Liang-Chun Shih, Theresa Staniczek, Tsu-Fang Wu, Hui-Ying Hsu, Wen-Dien Chang, Chih-Ho Lai, Chuan-Mu Chen

**Affiliations:** 1 Department of Otolaryngology-Head and Neck Surgery, China Medical University Hospital, Taichung 406, Taiwan; d22052121@gmail.com (Y.-A.T.); d6355@mail.cmuh.org.tw (C.-D.L.); minghsui5121@gmail.com (M.-H.T.); entdrshih7111@gmail.com (L.-C.S.); tfang_wu@yahoo.com.tw (T.-F.W.); 2 Department of Life Sciences, Ph.D. Program in Translational Medicine, National Chung Hsing University, Taichung 402, Taiwan; gary590422@yahoo.com.tw; 3 Center for Molecular Medicine, Graduate Institute of Biomedical Sciences, China Medical University, Taichung 406, Taiwan; proma1007@gmail.com (W.-C.C.); tennainging@gmail.com (H.-Y.H.); 4 Department of Dermatology, Venereology and Allergology, University Medical Center and Medical Faculty Mannheim, Center of Excellence in Dermatology, Heidelberg University, 68167 Mannheim, Germany; Theresa.Staniczek@medma.uni-heidelberg.de; 5 Department of Sport Performance, National Taiwan University of Sport, Taichung 404, Taiwan; changwendien@ntupes.edu.tw; 6 Department of Microbiology and Immunology, Graduate Institute of Biomedical Sciences, College of Medicine, Chang Gung University, Taoyuan 333, Taiwan; 7 Department of Pediatrics, Molecular Infectious Disease Research Center, Chang Gung Memorial Hospital, Linkou 333, Taiwan; 8 Department of Microbiology, School of Medicine, China Medical University, Taichung 406, Taiwan; 9 Department of Nursing, Asia University, Taichung 413, Taiwan; 10 The iEGG and Animal Biotechnology Center, Rong Hsing Research Center for Translational Medicine, National Chung Hsing University, Taichung 402, Taiwan

**Keywords:** hypopharyngeal squamous cell carcinoma, metformin, drug repositioning, anticancer, mortality, autophagy

## Abstract

Hypopharyngeal squamous cell carcinoma (HSCC) is usually diagnosed at an advanced stage, leading to a poor prognosis. Even after improvement of surgical techniques, chemotherapy, and radiation technology, the survival rate of HSCC remains poor. Metformin, which is commonly used for type 2 diabetes mellitus (DM), has been suggested to reduce the risk of various cancer types. However, only a few clinical studies mentioned the relationship between metformin use and HSCC. Hence, the aim of this study was to elucidate the specific effect and mechanism of action of metformin in hypopharyngeal cancer. We first assessed whether metformin use has an effect on hypopharyngeal cancer patients with DM by conducting a retrospective cohort study. Our results showed that DM hypopharyngeal cancer patients who used metformin exhibited significantly better overall survival rates than that without metformin treatment. The cell-based analysis further indicated that metformin treatment regulated p38/JNK pathway to reduce Cyclin D1 and Bcl-2 expressions. In addition, metformin activated the pathways of AMPKα and MEK/ERK to phosphorylate p27(Thr198) and reduce mTOR phosphorylation in cells. These actions direct cells toward G1 cell cycle arrest, apoptosis, and autophagy. Our results, through combining a clinical cohort analysis with an in vitro study, demonstrate that metformin can be used for drug repositioning in the treatment of DM patients with hypopharyngeal cancer.

## 1. Introduction

Patients with hypopharyngeal cancer exhibited the worst prognostic survival outcomes among all other head and neck cancer [1]. It is usually diagnosed at an advanced stage, resulting in a poor prognosis and a five-year survival rate as low as 30–35% [2]. Organ preservation therapy by concurrent chemoradiotherapy is the main treatment strategy in the first front-line therapy [3]. However, the treatment outcome varies and depends on intrinsic factors of patients including tumor nature (i.e., tumor, nodal stages, and differentiation) [4] or genetic background of patients’ condition [5]. The resistance to radiation and recurrence after therapy are the main drawbacks for patients with hypopharyngeal cancer, which result in a poor survival outcome [6,7]. This remains a point of emphasis for new strategies attempting to develop future treatments.

Metformin, a biguanide derivative, has been used to treat type 2 diabetes mellitus (T2DM) for nearly a century [8]. The benefits of metformin in diabetic patients are seen through reduced hepatic gluconeogenesis and increased muscle glucose uptake, thereby lowering the levels of insulin and glucose in the circulation [9]. Metformin also inhibits the synthesis of cholesterol, fatty acids, and proteins, while increasing the use of fatty acid [10]. In 2005, Evans was the first to describe the reduced risk of cancer in patients with T2DM treated with metformin [11]. This study led to extensive research on the antitumor activity of metformin [12]. Later studies reported that metformin decreased the risk of various types of cancer, including lung, breast, pancreas, liver, and colon cancer [13,14,15]. In addition, metformin use resulted in the prolongation of patient life and decreased cancer-related mortality [16]. Two mechanisms are involved in the antineoplastic activities of metformin: an “indirect effect”, leading to systemic changes in glucose or insulin levels, and a “direct effect” on tumor cells [17]. The direct anticancer effects of metformin are mediated through the activation of adenosine monophosphate-activated protein kinase (AMPK) and a decrease in mammalian target of rapamycin (mTOR) signaling, which suppressed the gluconeogenesis and protein synthesis [18].

Current therapeutic measures for hypopharyngeal carcinoma have many associated side effects that reduce the patient’s quality of life [19]. Therefore, identification of new and less cytotoxic treatments is urgently required. Metformin, used in T2DM, may decrease the risk of cancer [20]. There is only a clinical investigation recommends a correlation between metformin use and hypopharyngeal carcinoma, which has been documented in our recent study [21]. A previous study suggests that treatment of head and neck cancer with metformin via silencing long non-coding RNA (lncRNA) expression [22]. However, further studies are required to determine the mechanisms of action of metformin in the treatment of hypopharyngeal squamous cell carcinoma (HSCC). Thus, the aim of this study was to examine the effect of metformin use on DM patients with hypopharyngeal carcinoma. We also employed in vitro normal and tumor models of human cells to explore the molecular mechanisms of action of metformin in hypopharyngeal cancer.

## 2. Results

### 2.1. Metformin Increased Overall Survival in DM Patients with Hypopharyngeal Cancer

**Figure 1:**
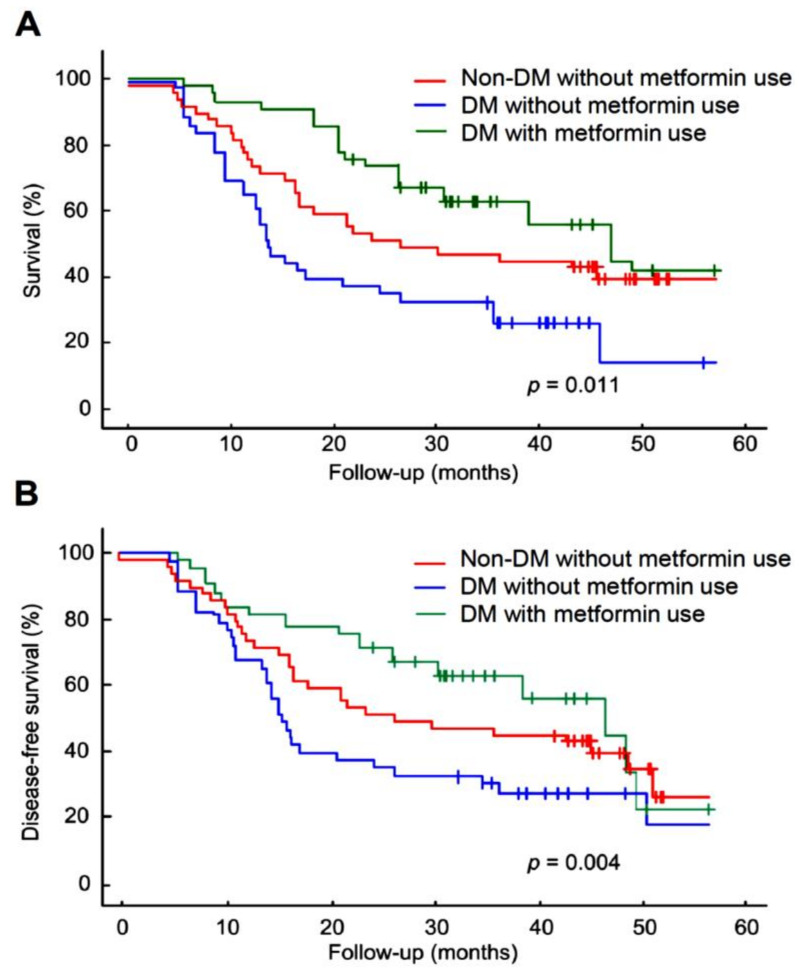
Kaplan–Meier analysis of (**A**) overall survival and (**B**) disease-free survival for patients with or without metformin treatment. A chi-squared test was performed to analyze the continuous variable between the groups. A *p*-value of less than 0.05 was considered statistically significant.

We first analyzed whether metformin prescription has an effect on DM patients with hypopharyngeal cancer. A total of 141 patients with hypopharyngeal cancer who underwent concurrent chemoradiotherapy were recruited in the study. There were 92 patients with DM and 49 hypopharyngeal cancer patients without DM. Of the 92 DM patients, 49 used metformin oral hypoglycemic agents (OHA) for DM control, and 43 patients used non-metformin OHA for DM control. The rates of overall survival and disease-free survival for the enrolled patients at 4 years were analyzed. The Kaplan–Meier analysis showed that the overall survival for non-DM without metformin use, DM without metformin use, and DM with metformin use were 40.8%, 27.9%, and 55.1% (*p* = 0.011), respectively (Figure 1A). In addition, the analysis for disease-free survival were non-DM without metformin use at 60.5%, DM without metformin use at 44.9%, and DM with metformin at 63.3% (*p* = 0.004) (Figure 1B). Compared to the control group of DM hypopharyngeal cancer patients without using metformin, the DM hypopharyngeal cancer patients with metformin treatment showed significantly better overall survival rates. These results indicate that metformin use is beneficial to increase the overall survival of hypopharyngeal cancer patients with DM.

### 2.2. Metformin Inhibited Cell Growth and Induced Cell Cycle Arrest in Hypopharyngeal Cancer Cells

**Figure 2:**
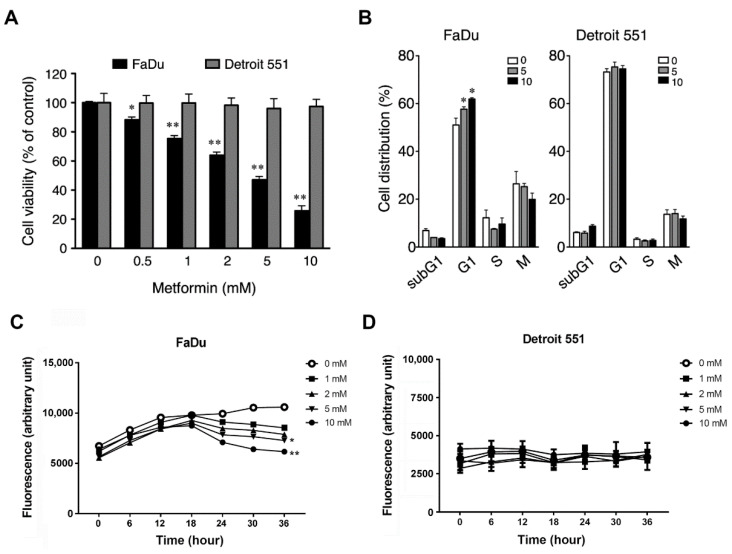
**Metformin inhibited the cell growth of hypopharyngeal cancer cells at G1 phase.** (**A**) Metformin inhibited the growth of FaDu (ATCC^®^ HTB-43™) but not Detroit 551 (ATCC^®^ CCL-110™) cells. Cell viability was measured by 3-(4,5-cimethylthiazol-2-yl)-2,5-diphenyl tetrazolium bromide (MTT) assay in triplicate. The viability of the culture, without metformin, was set as 100% alive. MTT assay was carried out after 48 h of metformin treatment. Statistical significance was evaluated by Student’s *t*-test. Black bars vs. gray bars (** *p* < 0.01; * *p* < 0.05). (**B**) Metformin caused cell cycle arrest of FaDu cells at G1 phase. FaDu and Detroit 551 cells were treated with metformin (0, 5, and 10 mM) for 48 h. Cell cycle profiles were analyzed in triplicate staining cells with propidium iodide. Metformin inhibited the cell proliferation of (**C**) FaDu but not (**D**) Detroit 551 cells. Cell proliferation assay was performed at the interval of 6 h incubation as mentioned in Section 4. Statistical significance was analyzed by Student’s *t*-test (** *p* < 0.01; * *p* < 0.05 vs. 0 mM).

To explore the functional mechanism of metformin in human hypopharyngeal tissues, and more specifically, to investigate whether metformin affects cell growth, we used FaDu (ATCC^®^ HTB-43™) and Detroit 551 (ATCC^®^ CCL-110™) cells as carcinoma and normal cell models, respectively. Our results showed that metformin treatment remarkably decreased the viability of FaDu cells in a dose-dependent manner, while barely influencing the growth of Detroit 551 cells (Figure 2A). We further examined the effect of metformin on cell proliferation. As shown in Figure 2C, FaDu cells without metformin treatment underwent normal cell proliferation during the culturing process. However, the cell proliferation was inhibited by metformin in the early stage (0–18 h), and then the cultures showed obvious cell death in the late stage (18–36 h) of metformin treatment dose-dependently. Compared to FaDu cells, Detroit 551 cells exhibited a slow and unaffected pattern of cell proliferation upon metformin treatment (Figure 2D). To verify which cell cycle stage was affected by metformin, we then performed flow cytometry to analyze the cell cycle distribution of FaDu and Detroit 551 cells following treatment with metformin. As shown in Figure 2B, the percentage of G1 phase cells increased by approximately 12% at the highest dose of metformin (10 mM) in FaDu but not in Detroit 551 cells. These results indicate that metformin effectively inhibited hypopharyngeal cancer cells by inducing cell cycle arrest at the G1 phase.

### 2.3. Metformin Downregulated Cyclin D1 and Bcl-2 and Induced Apoptosis

**Figure 3:**
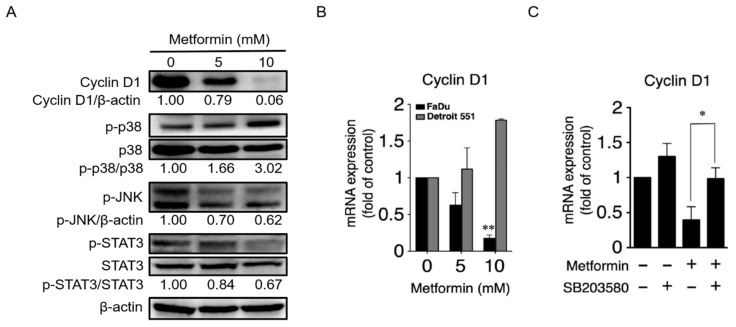
**Treatment with metformin suppressed Cyclin D1 expression in hypopharyngeal cancer cells.** (**A**) Metformin decreased Cyclin D1 expression. FaDu cells were treated with 0, 5, and 10 mM of metformin for 48 h. Cell lysates were separated by SDS-PAGE and analyzed by Western blot assay using the indicated antibodies. (**B**) Metformin decreased the mRNA expression of Cyclin D1 in hypopharyngeal cancer FaDu cells but not Detroit 551 cells. FaDu and Detroit 551 cells were treated with 0, 5, and 10 mM of metformin for 48 h. The mRNA levels of Cyclin D1 were analyzed by qRT-PCR. Statistical test compares the control group of 0 mM. (**C**) Treatment of the p38 inhibitor (SB203580) in metformin-treated FaDu cells leads to recovery in the levels of mRNA expression of Cyclin D1. Statistical significance indicates a comparison between groups (** *p* < 0.01; * *p* < 0.05 vs. 0 mM).

The interaction between cyclin-dependent kinases (Cdks) and their respective cyclins enables cells to progress through the G1 phase of the cell cycle [23,24]. Given that metformin resulted in cell cycle arrest at the G1 phase, we next investigated the protein expression level of Cyclin D1, a component of the cell cycle machinery that is necessary for the G1/S transition, in metformin-treated FaDu cells. Furthermore, we also investigated the possible role of MAPK signaling pathways on Cyclin D1 regulation [25,26]. As shown in Figure 3A, treatment of FaDu cells with metformin resulted in a strong, dose-dependent reduce in Cyclin D1 levels. Moreover, a decrease in phosphorylation of JNK and STAT3 was observed following an increased phosphorylation of p38 in cells treated with 10 mM metformin, suggesting that activated p38 downregulates phosphorylation of JNK (an activator of STAT3) and STAT3 (a transcription factor for Cyclin D1) [24]. A qRT-PCR analysis was then employed to detect mRNA expression levels of Cyclin D1 in metformin-treated FaDu and Detroit 551 cells. As shown in Figure 3B, the mRNA expression of Cyclin D1 was dramatically decreased with the highest dosage of metformin (10 mM) in FaDu cells. On the contrary, the mRNA expression levels of Cyclin D1 were increased in metformin-treated Detroit 551 cells. To further confirm the involvement of the p38/JNK/STAT3 signaling pathway on Cyclin D1 expression, we used SB203580, a p38 inhibitor, on metformin-treated FaDu cells and then assessed the levels of Cyclin D1 mRNA expression. Compared with the control, metformin-treated FaDu cells, use of SB203580 on metformin-treated FaDu cells led to a recovery in the levels of Cyclin D1 expression (Figure 3C).

**Figure 4:**
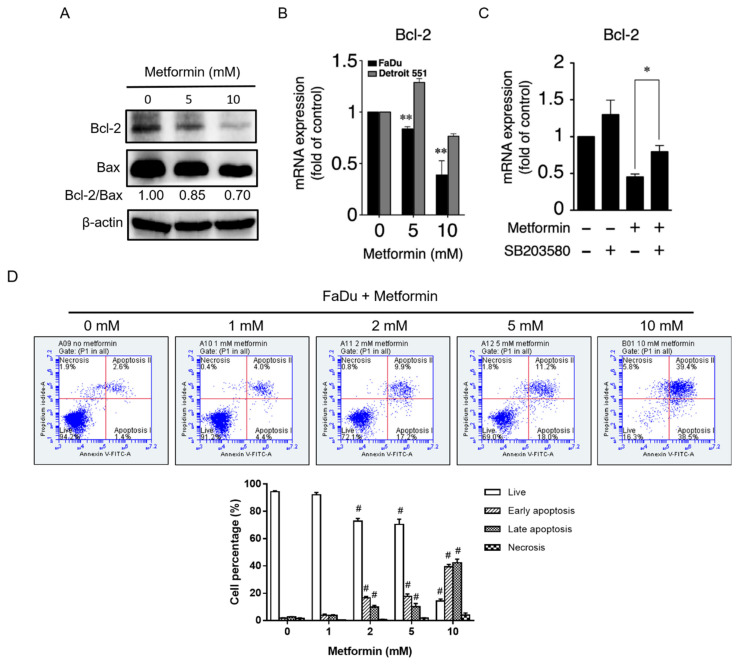
**Metformin inhibited Bcl-2 expression in hypopharyngeal cancer cells and induced apoptosis expression.** (**A**) FaDu cells were treated with 0, 5, and 10 mM of metformin for 48 h. Cell lysates were separated by SDS-PAGE and analyzed by Western blot assay with the indicated antibodies. (**B**) Treatment with metformin decreased the mRNA expression levels of Bcl-2 in FaDu but not in Detroit 551 cells. FaDu and Detroit 551 cells were treated with 0, 5, and 10 mM of metformin for 48 h. Bcl-2 mRNA levels were analyzed with qRT-PCR. Statistical significance indicates comparison analysis between the treatment and control group (0 mM). (**C**) Treatment with p38 inhibitor (SB203580) led to recovery of Bcl-2 mRNA expression levels in metformin-treated FaDu cells. (**D**) Treatment with metformin induces apoptosis of FaDu cells. Statistical significance was evaluated by Student’s *t*-test (* *p* < 0.05; ** *p* < 0.01; # *p* < 0.001 vs. 0 mM).

In addition, the protein and mRNA expression levels of Bcl-2 were remarkably reduced following treatment with the highest dose of metformin (10 mM) in FaDu cells (Figure 4A,B). The decrease in Bcl-2 might be due to upstream STAT3 inactivation [27]. To verify the role of the p38/JNK/STAT3 signaling pathway on regulation of Bcl-2 expression, we analyzed the effect of the p38 inhibitor SB203580 on Bcl2 expression in metformin-treated FaDu cells. As shown in Figure 4C, following treatment with SB203580, Bcl-2 mRNA expression recovered. These results revealed that metformin suppressed the expression of Cyclin D1 and Bcl-2 mediated through regulation of p38, JNK, and STAT3 signaling pathway. Because of the antiapoptotic effect of Bcl-2 and the reduction of Bcl-2/Bax ratio, we examined whether metformin induced an apoptosis in FaDu cells. As shown in Figure 4D, metformin treatment directed FaDu cells toward significant apoptosis. At 10 mM of metformin, approximately 80% of cells underwent apoptosis.

### 2.4. Treatment of Hypopharyngeal Cancer Cells with Metformin Also Induced Autophagy

**Figure 5:**
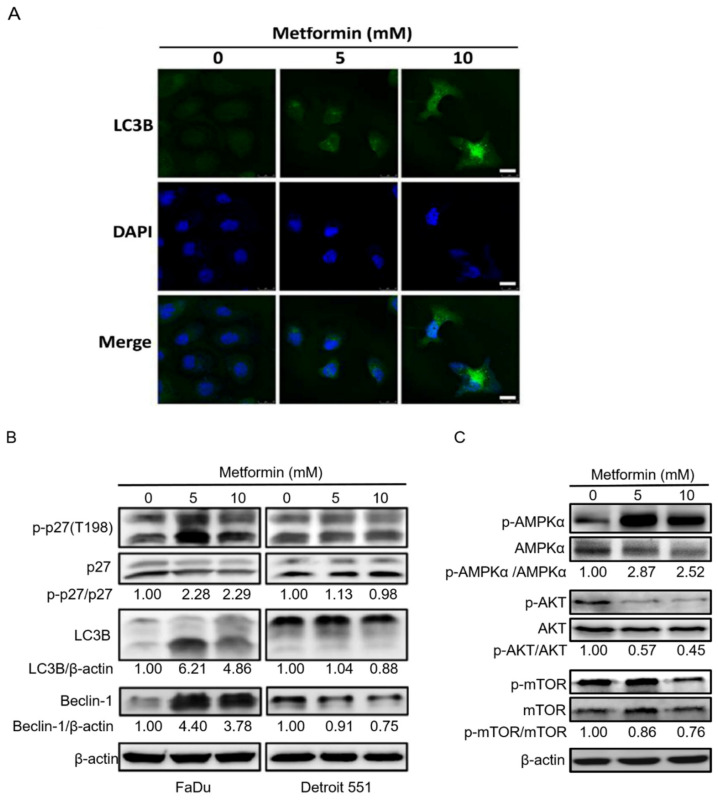
**Metformin induced autophagy in hypopharyngeal cancer cells.** (**A**) Metformin increased LC3B expression. FaDu cells were treated with 0, 5, and 10 mM metformin for 48 h. The endogenous LC3B expression was assessed by confocal microscopy after staining with anti-LC3B antibodies (green) and 4′,6-diamidino-2-phenylindole (DAPI) (blue). Scale bars, 25 µm. (**B**) Metformin upregulated p27(Thr198) phosphorylation and expression of LC3B and Beclin-1. FaDu and Detroit 551 cells were treated with 0, 5, 10 mM metformin for 48 h. Cell lysates were separated by SDS-PAGE and analyzed by Western blot with the indicated antibodies. (**C**) Metformin reduced mTOR phosphorylation via AMPKα phosphorylation in hypopharyngeal cancer cells. FaDu cells were treated with 0, 5, and 10 mM metformin for 48 h. Cell lysates were separated by SDS-PAGE and analyzed by Western blot assay.

The cytoprotective function of Bcl-2 proteins stems from their ability to antagonize Bax and Bak, and thus prevent apoptosis. Consequently, loss of Bcl-2 in cells has been shown to activate autophagy [28]. Phospho-p27(T198) leads to stabilization of p27, which allows cells to survive during growth factor withdrawal and metabolic stress environment by inducing autophagy [29]. Both LC3B and Beclin-1 are used as markers of autophagy, and more specifically, as markers of vesicle nucleation and elongation, respectively [30]. Our results showed that following metformin treatment, LC3B expression was increased in FaDu cells, and the levels of LC3B, Beclin-1, and the phosphorylation of p27(Thr198) were raised in FaDu but not in Detroit 551 cells, suggesting the activation of autophagy by metformin occurred in hypopharyngeal cancer FaDu cells (Figure 5A,B). In particular, at 10 mM of metformin, the treatment caused the accumulation of LC3B abundantly in the cytoplasm of FaDu cells, indicating autophagosomes were generated at multiple sites throughout the cytoplasm (Figure 5A). Moreover, metformin also caused obvious changes in the morphology of FaDu cells from epithelial-like to swollen and irregular shape. We then explored the specific mechanisms of autophagy present in metformin-treated FaDu cells by investigating classical pathways, such as AMPK/mTOR and autophagy-related proteins. As shown in Figure 5C, metformin induced AMPK activation to suppress mTOR phosphorylation and block AKT phosphorylation.

### 2.5. Metformin Inhibited Hypopharyngeal Cancer Cells via the MEK/ERK/RSK Signaling Pathways

**Figure 6:**
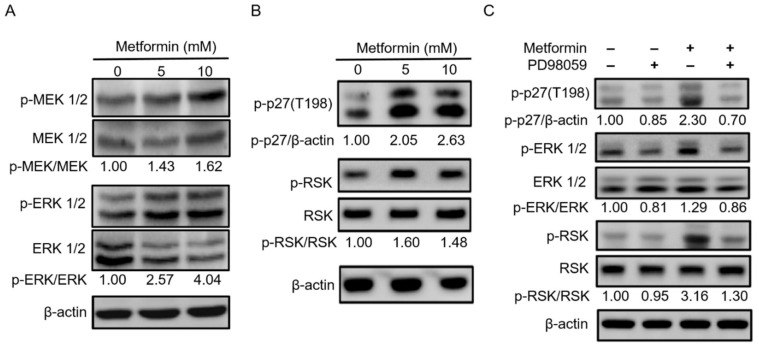
**Treatment of hypopharyngeal cancer cells with metformin triggered p27(T198) phosphorylation.** (**A**,**B**) Metformin induced phosphorylation of MEK1/2, ERK1/2, p27(T198), and downstream RSK. FaDu cells were treated with 0, 5, and 10 mM metformin for 48 h. Cell lysates were analyzed using Western blot assay. (**C**) The MEK/ERK inhibitor (PD98059) decreased phosphorylation of p27(T198) in metformin-treated hypopharyngeal cancer cells. FaDu cells were treated with 5 mM metformin for 48 h. Cell lysates were analyzed by Western blot assay. The relative protein band intensities were quantitated by ImageJ software and normalized by the internal control of β-actin.

Inhibitor of mTOR leads to reassociation of dephosphorylated Atg13 with Atg1, stimulating its catalytic activity and inducing autophagy [31]. The major function of activation of MEK1/2 and ERK1/2 signaling pathway is correlated with cell proliferation [24]. However, we had an opposed result and suggested that another mechanism occurred in FaDu cells on the basis of metformin inhibiting the growth of hypopharyngeal cancer cells (Figure 6A). A previous study showed that activated AMPK or RSK1 resulting in phosphorylation of p27 at Thr198 is strongly expressed in quiescent cells [32]. Given that RSK1 is downstream of MEK/ERK signaling pathway, we then examined whether the MEK/ERK/RSK signaling axis is involved in p27(Thr198) phosphorylation. As shown in Figure 6B, metformin-induced phosphorylation of RSK increased p27(T198) phosphorylation in treated FaDu cells. To further confirm whether MEK/ERK/RSK signaling pathway is required for p27(T198) phosphorylation, we used PD98059, an inhibitor of ERK. Our results showed that indeed PD98059 blocked ERK and downstream RSK phosphorylation to decrease p27(T198) phosphorylation in metformin-treated hypopharyngeal cancer FaDu cells (Figure 6C). These results from cell-based studies demonstrate that metformin induced hypopharyngeal cancer cell death, which is mediated by an autophagy mechanism associated with the AMPKα/mTOR and MEK/ERK/RSK signaling pathways.

## 3. Discussion

**Figure 7:**
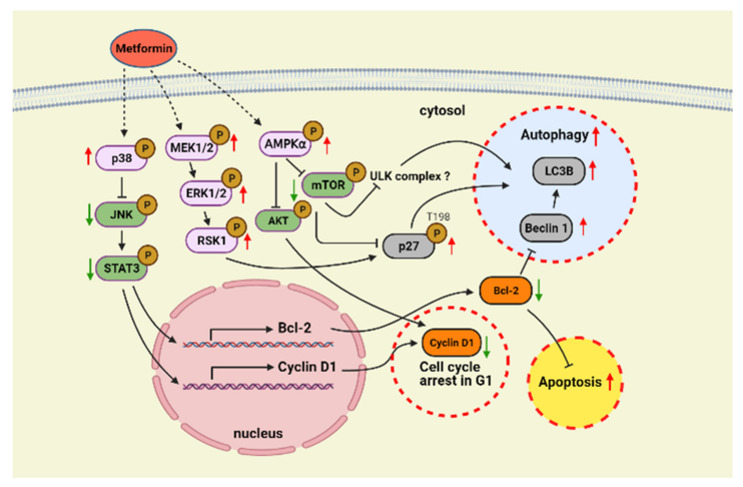
**Schematic showing potential mechanisms of autophagy and apoptosis induction in metformin-treated hypopharyngeal cancer cells.** Metformin induces p38 activation to decrease JNK/STAT3 phosphorylation, resulting in reduction in Cyclin D1 and Bcl-2. Loss of Cyclin D1 causes cell cycle arrest at the G1 phase. Downregulation of Bcl-2 may cause the activation of both apoptosis and autophagy. In addition, metformin activates AMPKα and MEK/ERK/RSK signaling pathways to reduce mTOR and increase p27(Thr198) phosphorylation; these events may direct hypopharyngeal cancer cells toward autophagy. Upward red arrows indicate that the expression or phosphorylation of target proteins were upregulated by metformin treatment, while downward green arrows indicate the opposite situation.

In this study, we showed that metformin use increased overall survival in hypopharyngeal cancer patients with DM (Figure 1). The possible mechanism of metformin in the treatment of hypopharyngeal cancer cells was also investigated. Apoptosis and autophagy are two common types of cell death, in which some effector molecules and signal pathways are closely associated with each other. The metformin-mediated cell death may direct cancer cells to either apoptosis or autophagy, or the combination of both [27,33]. We demonstrated that metformin activates p38 to inhibit the phosphorylation of JNK and STAT3, leading to a decrease in expression of cyclin D1 and Bcl-2 at both mRNA and protein levels (Figure 3 and Figure 4). Downregulation of cyclin D1 caused cell cycle arrest at G1 phase (Figure 2). Bcl-2 is known as its antiapoptotic role in many cases [34]. The downregulation of Bcl-2 by metformin treatment was linked with an increased apoptosis in present hypopharyngeal cancer cells. Meanwhile, Bcl-2 is reported to inhibit autophagy by complexing with Beclin-1 [35]. Therefore, we proposed that the reduction of Bcl-2 may potentiate the occurrence of autophagy. This was confirmed by increased Beclin-1 and LC3B protein levels in hypopharyngeal cancer cells treated with metformin (Figure 5). In addition, metformin induced p27(Thr198) phosphorylation through activation of MEK/ERK/RSK and AMPK/mTOR signaling pathways (Figure 6), and consequently directed hypopharyngeal cancer cells toward autophagy (Figure 7). Metformin also induces autophagy through the activation of AMPK/mTOR pathway in multiple myeloma [36] and breast cancer [37], also in other cell types and animal models [38]. These accumulated results suggest that the metformin directs hypopharyngeal cancer cells toward autophagy (Figure 7).

This study used Detroit 551 cells as a normal cell control, which has been used for cancer-normal comparison in some anticancer studies, including breast cancer [39], ovarian cancer [40], lung cancer [41], and head and neck cancer [42]. Compared to the significant decrease in cell viability observed in hypopharyngeal cancer FaDu cells, Detroit 551 cells only showed a 3% decrease following treatment with 10 mM metformin (Figure 2). Consistent results were observed in the proliferation assay, where metformin inhibited the proliferation of FaDu cells dose-dependently but caused no effect on Detroit 551 cells. Results of cell cycle analysis then showed that metformin did not affect the cell cycle progression in Detroit 551. Although earlier studies showed that metformin can reduce the proliferation of normal dermal fibroblasts [43], the phenomenon cannot be found in Detroit 551 cells. This may be due to different characteristics of cell lines or caused by a shorter culturing time in our experiments (36–48 h vs. 120 h).

Metformin-mediated cell cycle arrest has been found in many cancer types. Under normal circumstances, the cell cycle is tightly regulated by the interaction of different cyclins and cyclin-dependent kinases (CDKs) at multiple checkpoints. The cyclin D1-CDK4 complex is an important checkpoint for the progression of cell cycle through G1 phase. Our studies confirmed that treatment with metformin resulted in loss of Cyclin D1, which in turn caused cell cycle arrest at G1 phase (Figure 2B). We further indicated that the p38/JNK/STAT3 pathway regulates levels of Cyclin D1 expression. Studies indicated that AMPK activation may inhibit AKT signaling pathway, subsequently leading to cyclin D1 reduction [44]. This was also observed in our study and may contribute to explain the reduction of cyclin D1. In addition, treatment of esophageal squamous cell carcinoma with metformin was shown to downregulate Bcl-2 expression through STAT3 inactivation in a previous study [28]. We also showed that activation of p38 leads to downregulation of JNK and its downstream target STAT3, leading to decreased expression of Bcl-2, which in turn, results in inhibition of cell growth and induction of apoptosis and autophagy in metformin treated-hypopharyngeal cancer FaDu cells. The antiapoptotic function of Bcl-2 is based on its binding with Bax protein to block the formation of pro-apoptotic Bax/Bax homodimer [45]. The intracellular ratio of Bcl-2/Bax determines whether apoptosis was initiated. In this study, metformin treatment reduced intracellular Bcl-2 level and Bcl-2/Bax ratio, and also significantly increased the ratio of apoptotic cell population (Figure 4). On the other hand, Bcl-2 inhibits autophagy by forming Bcl-2/Beclin-1 heterodimer [35]. Therefore, as intracellular Bcl-2 decreased by metformin, it will reduce the chance of Beclin-1 to form dimer with Bcl-2 or increase the chance of Beclin-1 to be released from Bcl-2/Beclin-1 dimer to promote the progress of autophagy.

The metformin induced-metabolic stress leads to a high AMP/ATP ratio, which triggers AMPK activation [46]. AMPK activation results in energy preservation and increased production of ATP [47]. Most importantly, AMPK activation also induces p53 phosphorylation, which initiates AMPK-dependent cell cycle arrest [48]. Due to the presence of a TP53 gene mutation in FaDu cells, we did not consider p53 to be involved in this process. The role of AMPK in metabolic regulation is well understood. More recently, AMPK becomes a possible tumor suppressor and target for cancer prevention and treatment. Many studies demonstrated that AMPK can regulate autophagy via regulating downstream signaling molecules, such as inhibiting mTORC1, activating ULK1 complex, and activating SNARE-like proteins [49]. Metformin-mediated autophagy via the activation of AMPK/mTOR pathways were found in multiple myeloma [36] and breast cancer [37], and also in other cell types and animal models [38]. In the present study, metformin directed hypopharyngeal cancer cells toward autophagy. This is evidenced by increased Beclin-1 and LC3B protein levels, which are two essential proteins for autophagy initiation and autophagosomes formation. Moreover, as an important central energy sensor, a series of studies demonstrate that AMPK activation also involves with the progression of apoptosis [44]. In fact, the precise molecular mechanisms about the connection of metformin-mediated AMPK activation with cell death (cell cycle arrest, apoptosis, autophagy) in various cancer cells have not been fully elucidated.

MEK1/2 and ERK1/2 signaling pathways possess major roles in cell proliferation [24]. However, metformin inhibits the growth of hypopharyngeal cancer cells even under activation of this pathway, which is why we considered p27 and possible downstream activated factors in the current study. p27 is regarded as a proliferative inhibitor in various malignant tumor cells [50]. In recent years, new functions of p27 have been reported, such as the regulation of autophagy [51]. Phosphorylation of p27(Thr198) mediates induction of autophagy through its stability [30]. Recently, metformin was found to inhibit angiogenic capacity of endothelial progenitor cells via increasing p27 phosphorylation and AMPK-mediated autophagy pathway [52].

Since the first report in 2005, metformin has the potential role to extend the life span of patients with diabetic cancer [11]. In the past decade, many studies have indicated the effect of metformin in inhibiting the growth of clinical cancers [13,14,15]. The underlying anticancer mechanisms have been elucidated in various cancer cell models. However, the available information of the effect of metformin on hypopharyngeal cancer cells remains limited. To date, only one paper has reported that metformin inhibits FaDu cell proliferation by epigenetically decreasing long non-coding RNA SNHG7 [22]. To the best of our knowledge, there are no published findings showing that metformin induces both apoptosis and autophagy in hypopharyngeal cancer cells, and the relationship of p27(Thr198) phosphorylation and autophagy is the first present in diverse anticancer mechanisms of metformin. There are some limitations in the present study, including the crosstalk between diverse signaling pathways, downstream molecules of AMPK-dependent and AMPK-independent signaling pathways, and central regulators of autophagy and apoptosis. Nevertheless, the present study provides useful information for the application of metformin in treating hypopharyngeal cancer and also provides directions to investigate the underlying mechanisms of metformin in hypopharyngeal cancer in the near future.

## 4. Materials and Methods

### 4.1. Patient Selection and Data Collection

From 1 December 2011, to 31 December 2013, a total of 141 patients with hypopharyngeal cancer were enrolled in the cohort study. Demographic factors of patients including age, gender, alcohol use, betel nut chewing, and cigarette smoking were documented. Metformin use was based on their previous oral hypoglycemic agents (OHA) administration and persisted through the concurrent chemoradiotherapy (CCRT) treatment until the latest follow-up. The minimal follow-up duration was 4 years. The clinical TNM (tumor, node, metastasis) stage, survival outcome, and disease control were recorded as parameters for further analysis. This study was approved by the Institutional Review Board of China Medical University Hospital, a 2500-bed tertiary teaching hospital in central Taiwan (no. CMUH103-REC1–078). All enrolled patients provided informed consent after a complete explanation of the study protocol. All the clinical data were collected by medical chart review for the patients with hypopharyngeal cancer.

### 4.2. Antibodies

Monoclonal antibodies against phospho-AMPKα(thr172), phospho-STAT3(tyr705), phospho-ERK1/2(thr202/tyr204), phospho-p38(thr180/tyr182), p38, phospho-MEK1/2(ser221), MEK1/2, phospho-AKT(ser473), AKT, Bcl-2, Bax, p27, and polyclonal antibodies against phospho-mTOR(ser2448), mTOR, AMPKα, phospho-RSK(Thr359/Ser363), RSK, ERK1/2, LC3B, and Beclin 1 were purchased from Cell Signaling (Cell Signaling Technology, Inc., Beverly, MA, USA). Monoclonal antibodies specific to phospho-JNK(thr183/tyr185) and JNK, and polyclonal antibodies specific to STAT3 were purchased from Santa Cruz (Santa Cruz Biotechnology, Inc., Santa Cruz, CA, USA). Monoclonal antibody against Cyclin D1, polyclonal antibody against β-actin, and horseradish peroxidase (HRP)-conjugated anti-rabbit and anti-mouse secondary antibodies were purchased from Abcam (Burlingame, CA, USA). Anti-phospho-p27(Thr198) polyclonal antibody was purchased from R&D (R&D Systems, Inc., Minneapolis, MN, USA).

### 4.3. Cell Culture and Treatment

Human hypopharyngeal cancer FaDu cells (ATCC^®^ HTB-43™) and human skin fibroblast Detroit 551 cells (ATCC^®^ CCL-110™) were cultured in Eagle’s minimum essential medium (Thermo Fisher Scientific, GIBCO, Waltham, MA, USA) supplemented with 10% fetal bovine serum (HyClone, UT, USA), 1% penicillin–streptomycin (Thermo Fisher Scientific, GIBCO, Waltham, MA, USA), 1% non-essential amino acid, and 1% sodium pyruvate at 37 °C in an atmosphere containing 5% CO_2_. Cells were harvested after 48 h in metformin (Sigma-Aldrich, St. Louis, MO, USA) treatment. For protein expression analysis of signaling pathways, the cells were pretreated with the inhibitors of PD98059 (Calbiochem, San Diego, CA, USA) or SB203580 (Calbiochem, San Diego, CA, USA) for 1 h.

### 4.4. Cell Viability and Proliferation Assay

FaDu (5 ×10^4^ cells per well) and Detroit 551 cells (1.25 ×10^4^ cells per well) were grown in 24-well plates and treated with metformin (0, 5, 10 mM). After 48 h, cells were refreshed with 0.5 mL of media containing 0.5 mg/mL of 3-(4,5-cimethylthiazol-2-yl)-2,5-diphenyl tetrazolium bromide (MTT; Sigma-Aldrich, St. Louis, MO, USA) and cultured for 1 h. The culture media was discarded and 1 mL of dimethyl sulfoxide (DMSO; Sigma-Aldrich) was added for 10 min. One hundred microliters of supernatant were transferred into a 96-well plate for measuring absorbance on an ELISA reader at 570 nm, as described previously [53]. Cell proliferation was measured using the Deep Blue Cell Viability Kit (BioLegend, San Diego, CA, USA), which measures the reduction of reazurin to fluorescent resorufin present in the culture. Initially, FaDu (1 × 10^4^ cells per well) and Detroit 551 (2500 cells per well) cells in 100 μL culture medium with metformin (0, 1, 2, 5, 10 mM) were grown on 96-well plates. For the measurement of cell proliferation, 10 μL of Deep Blue Cell Viability Kit was added onto each well and incubated at 37 °C for 2 h; then, the fluorescence (excitation at 530 nm, emission at 590 nm) was measured using a microplate reader. Afterward, the medium with metformin (0, 1, 2, 5, 10 mM) was refreshed and the incubation was continued. Data are presented as the amount of fluorescence of cells versus the time duration of metformin treatment.

### 4.5. Cell Cycle Analysis

FaDu or Detroit 551 cells were treated with metformin (0, 5, 10 mM) for 48 h. Cells were prepared and fixed in ice-cold methanol. On the day of analysis, methanol was removed, and the cells were stained with a propidium iodide (PI) solution (10 μg/mL PI, 100 μg/mL RNase A, and 0.1% *v/v* Triton X-100 in phosphate buffered saline (PBS)) for 30 min. Cell suspensions were filtered and transferred into a flow cytometer (FACScalibur; Becton Dickinson, Franklin Lakes, NJ, USA) for measuring cell fluorescence (PI excitation at 536 nm; emission at 617 nm). Results were analyzed with the Flowjo V9.7.5 software (TreeStar Inc., Ashland, OR, USA) to evaluate the percentages of cells in G0/G1, S, and G2/M phases, as described previously [54].

### 4.6. Apoptosis Assay

Apoptosis assay was performed using a fluorescein (FITC) Annexin V Apoptosis Detection Kit (Cat. No. 556547, BD Pharmingen Inc., San Diego, CA, USA). FaDu cells were treated with metformin (0, 1, 2, 5, 10 mM) for 48 h. At the end of treatment, cells were washed twice with cold PBS and then resuspended in 1X binding buffer (10 mM HEPES (N-2-hydroxyethylpiperazine-N-2-ethane sulfonic acid)/NaOH (pH 7.4), 140 mM NaCl, 2.5 mM CaCl_2_) at a concentration of ≈1 ×10^6^ cells/mL. Then, 100 μL of cell solution was mixed with 5 μL of FITC-labeled annexin V antibody and 5 μL of PI. The cells were gently vortexed and then incubated for 15 min at room temperature in the dark. A total of 400 μL of 1X binding buffer was added to dilute the cell solution. Samples were filtered and analyzed by flow cytometer (Accuri C6 Plus, BD Biosciences, Franklin Lakes, NJ, USA).

### 4.7. Quantitative Real-Time Reverse Transcription-PCR (qRT-PCR)

qRT-PCR was employed to determine the mRNA expression for Cyclin D1 and Bcl-2. Total mRNA was prepared from treated FaDu and Detroit 551 cells by RNAlater Stabilization Solution (ThermoFisher Scientific). Reverse transcription was performed using PrimeScript RT Master Mix (Takara, Shiga, Japan). qRT-PCR assay was performed using 20 μL of a reaction mixture that contained 10 ng of complementary DNA (cDNA), primers, and SYBR Premix Ex Taq (Takara, Shiga, Japan). The relative expression level was calculated using the 2^−ΔΔCt^ method with glyceraldehyde 3-phosphate dehydrogenase (GADPH) as the internal control, and the difference was regarded as significant only when the absolute value of ΔΔCt ≥ 1. The primer sequences for qRT-PCR were corresponded to human Cyclin D1 (forward, 5′-CCCTGACGGCCGAGAAG-3′; and reverse, 5′-AGGTTCCACTTGAGCTTGTTCAC-3′), Bcl-2 (forward, 5′-ACCTGCACACCTGGATCCA-3′; and reverse, 5′-AGAGACAGCCAGGAGAAATCAAA-3′), and GAPDH (forward, 5′-CCCCCAATGTATCCGTTGTG-3′; and reverse, 5′-TAGCCCAGGATGCCCTTTAGT-3′).

### 4.8. Western Blot Assay

Whole cell lysates were prepared, and total proteins were separated by sodium dodecyl sulfate polyacrylamide gel electrophoresis (SDS-PAGE) and transferred to polyvinylidene difluoride membranes (Millipore, Burlington, MA, USA). The blots were incubated with 5% skim milk at room temperature for 1 h, then stained with primary antibodies, and then incubated with peroxidase-conjugated secondary antibody. The proteins of interest were detected by ECL Western blotting detection reagents (GE Healthcare, Piscataway, NJ, USA). The protein expression levels were analyzed using an Azure c400 system and AzureSpot Analysis Software (Azure Biosystems, Dublin, CA, USA) following the manufacturer’s instructions [55].

### 4.9. Immunofluorescence Staining and Confocal Laser Scanning Microscopy

FaDu cells were grown on glass coverslips in 6-well plates and treated with 0, 5, or 10 mM metformin for 48 h. The treated cells were fixed with 4% formaldehyde for 10 min and permeabilized with 0.1% Triton X-100 in PBS at 4 °C for 10 min. The samples were then blocked with 3% bovine serum albumin (BSA) in PBS at 37 °C for 30 min, then stained with anti-LC3B (Cell Signaling Technology no. 3868; diluted 1:200) and Alexa Fluor 488-conjugated anti-mouse antibody (Jackson ImmunoResearch Laboratories, West Grove, PA, USA; diluted 1:400). 4′,6-diamidino-2-phenylindole (DAPI) (0.2 μg/mL) was used for nuclear staining. The stained cells were then analyzed under a confocal microscopy (Leica TCS SP8X laser-scanning microscope using a 63 × 1.4 numerical aperture objective) [56].

### 4.10. Statistical Analysis

Date from primary diagnosis to recurrence or death was recorded as disease-free survival (DFS), and date from primary diagnosis to last documented note or death was recorded as overall survival (OS). Kaplan–Meier analysis was performed to assess DFS and OS values. Univariate analysis was performed using a Cox proportional hazards model. For between-group comparisons, continuous variable was performed using a chi-squared test. For cell-based studies, the results were calculated from 3 independent experiments and analyzed by using Student’s *t*-test. The analyses were conducted using SAS statistical software (Version 9.3 for Windows; SAS Institute, Inc., Cary, NC, USA). A *p*-value of less than 0.05 was considered statistically significant.

## 5. Conclusions

These results revealed that DM hypopharyngeal cancer patients who used metformin exhibited significantly better overall survival rates than that without metformin treatment. The in vitro cell-based studies demonstrated that metformin induced cell cycle arrest, apoptosis, and autophagy in hypopharyngeal cancer cells. The results from this study provide insights into the mechanisms by which metformin inhibits hypopharyngeal cancer and may also be used for drug repurposing of metformin as a potent therapeutic agent for DM patients with hypopharyngeal cancer.
